# OrgaMapper: a robust and easy-to-use workflow for analyzing organelle positioning

**DOI:** 10.1186/s12915-024-02015-8

**Published:** 2024-09-30

**Authors:** Christopher Schmied, Michael Ebner, Paula Samsó, Rozemarijn Van Der Veen, Volker Haucke, Martin Lehmann

**Affiliations:** 1 Leibniz-Forschungsinstitut Für Molekulare Pharmakologie (FMP), Robert-Roessle-Straße 10, Berlin, 13125 Germany; 2 Present address: EU-OPENSCREEN ERIC, Robert-Roessle-Straße 10, Berlin, 13125 Germany; 3 Department of Biology, Chemistry, Pharmacy, Freie Universität Berlin, Berlin, 14195 Germany

**Keywords:** ImageJ, Fiji, Segmentation, Image analysis, Data analysis, R, Shiny, Organelle position

## Abstract

**Background:**

Eukaryotic cells are highly compartmentalized by a variety of organelles that carry out specific cellular processes. The position of these organelles within the cell is elaborately regulated and vital for their function. For instance, the position of lysosomes relative to the nucleus controls their degradative capacity and is altered in pathophysiological conditions. The molecular components orchestrating the precise localization of organelles remain incompletely understood. A confounding factor in these studies is the fact that organelle positioning is surprisingly non-trivial to address e.g., perturbations that affect the localization of organelles often lead to secondary phenotypes such as changes in cell or organelle size. These phenotypes could potentially mask effects or lead to the identification of false positive hits. To uncover and test potential molecular components at scale, accurate and easy-to-use analysis tools are required that allow robust measurements of organelle positioning.

**Results:**

Here, we present an analysis workflow for the faithful, robust, and quantitative analysis of organelle positioning phenotypes. Our workflow consists of an easy-to-use Fiji plugin and an R Shiny App. These tools enable users without background in image or data analysis to (1) segment single cells and nuclei and to detect organelles, (2) to measure cell size and the distance between detected organelles and the nucleus, (3) to measure intensities in the organelle channel plus one additional channel, (4) to measure radial intensity profiles of organellar markers, and (5) to plot the results in informative graphs. Using simulated data and immunofluorescent images of cells in which the function of known factors for lysosome positioning has been perturbed, we show that the workflow is robust against common problems for the accurate assessment of organelle positioning such as changes of cell shape and size, organelle size and background.

**Conclusions:**

OrgaMapper is a versatile, robust, and easy-to-use automated image analysis workflow that can be utilized in microscopy-based hypothesis testing and screens. It effectively allows for the mapping of the intracellular space and enables the discovery of novel regulators of organelle positioning.

**Supplementary Information:**

The online version contains supplementary material available at 10.1186/s12915-024-02015-8.

## Background

Eukaryotic cells are highly compartmentalized by membrane-enclosed and membrane-less organelles. Compartmentalization of the cytoplasm into the semi-confined spaces of specialized organelles ensures the efficient channeling of all fundamental biological processes that are hallmarks of eukaryotic life. The position of these organelles within the cytoplasm is highly specific, subject to regulation by metabolic cues and signaling pathways, and interdependent with organelle identity and function [1, 2]. The wide range of human diseases that are associated with organelle transport is testimony to the paramount importance of the fidelity of organelle positioning [2–4]. The transport and dynamic positioning of organelles is governed by specialized molecular motors as most dramatically exemplified in highly polarized cells such as neurons [5]. These molecular motors use the actin and microtubule cytoskeleton as tracks and a plethora of scaffolding, regulatory, and signaling proteins to connect to specific cargo organelles. More recently, membrane contact sites between different organelle species were uncovered as an additional layer in the complex regulatory network that coordinates organelle positioning [6].

Molecular mechanisms and functional relevance of organelle positioning are the focus of intense research with examples ranging from early endosomes [7, 8] recycling endosomes [9] autophagosomes [10, 11], peroxisomes [12, 13], lipid droplets [14], mitochondria [15–17], to the Golgi complex [18, 19]. Notably, in the last decade, the positioning of lysosomes and late endosomes has specifically gained attention as it is inextricably linked to metabolic homeostasis and growth signaling [20, 21]. Even the intracellular distribution and function of membrane-less organelles such as stress granules is regulated by microtubule-based transport [22, 23]. In patient-derived cells, altered organelle positioning can hint at possible molecular underpinnings of disease or provide cues regarding putative disease mechanisms [24, 25].

Evidently, there is a recent surge in efforts to decipher the physiological cues and molecular components that govern organelle positioning in health and disease and many novel cell biological concepts and disease links were uncovered on the way. However, our understanding of these processes is far from complete. For instance, it is mostly unclear which factors on organelle membranes bridge to the molecular motor machinery, how and via which factors organelles signal to the machinery, and how the transport machinery is instructed by external cues. In order to identify factors involved in these mechanisms and to facilitate the use of organelle positioning as a diagnostic readout [24], biologists and pathologists require easy-to-use and scalable tools. These tools must be able to faithfully and robustly localize organelles in images and provide easily interpretable measurements and analysis.

There are many general-purpose solutions and platforms available, such as Fiji, Icy, Cell Profiler, Knime, or Illastik, that enable scientists to implement image analysis workflows [26–30]. However, creating a robust and easy-to-use analysis workflow for complex biological phenomena such as organelle positioning requires specialized expertise in image analysis and software engineering. To allow biologists with little to no background in programming and data science to execute advanced image and data analysis, well-tested workflow templates are required that use established components, are provided to the user with comfortable graphical user interfaces (GUIs), and are supported with ample documentation [31]. Eventually, such workflows will promote the transparent and reproducible publication of images and the results of image analysis [32]. Fortunately, this need has become recognized in the scientific community, creating the emerging field of bioimage analysis [33] with such workflow templates being increasingly published and made available to the broader community [34–37].

We reviewed published approaches to quantify organelle positioning and encountered numerous different strategies, most of them based on manual or semi-automatic custom-made solutions either using intensity or the distance of individual objects as measurement readout (Additional File 1, Additional File 2). Here, we addressed the advantages and shortcomings of different basic analysis strategies using simulated data. We found that organelle positioning is surprisingly non-trivial to address as it is highly sensitive to cell size, cell shape, organelle shape, background intensity, or organelle intensity distributions. Based on these findings we implemented detection- and intensity-based analysis in a complete open source image and data analysis workflow as an ImageJ/Fiji Plugin and an easy-to-use R Shiny App called OrgaMapper. This workflow enables users with little background in image and data analysis to robustly quantify organelle positioning while accounting for confounding factors in the datasets, such as differences in cell size or organelle shape. We validated the analysis workflow using real-world immunofluorescence datasets and established organelle positioning phenotypes. The entire workflow, accompanying test data, and extensive documentation are open source and freely available to the community. We thus provide the rapidly expanding field of organelle positioning with a publicly accessible, validated, fully automated, and easy-to-use analysis workflow.

## Results

### Distance of individual organelles to the nucleus is a robust readout of organelle positioning

**Fig. 1:**
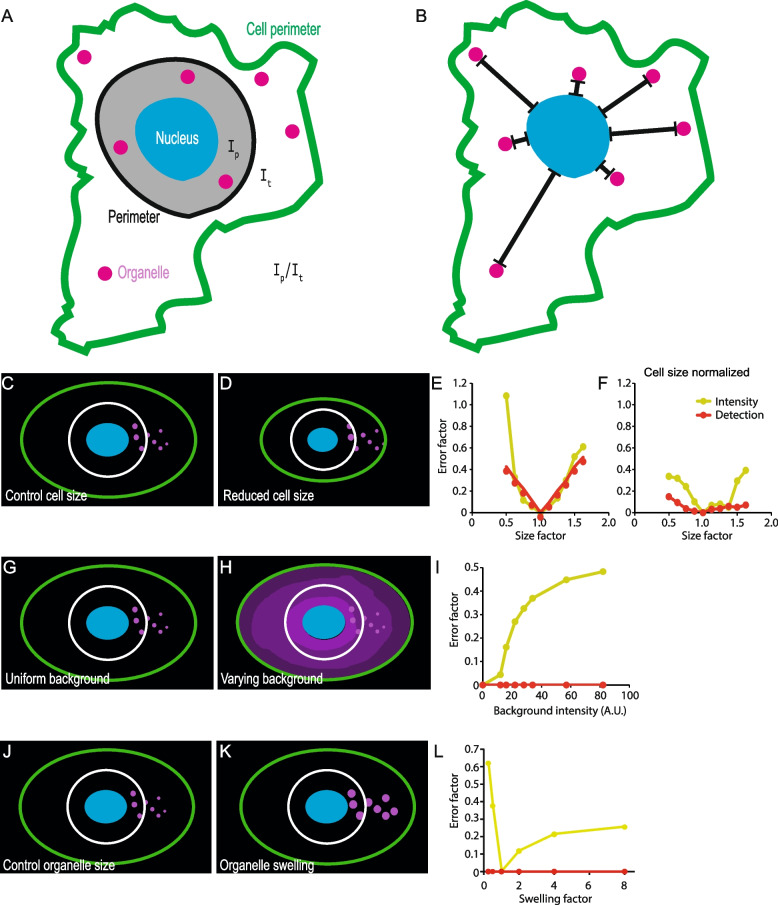
Measuring the distance of individual organelles to the nucleus is robust to changes in cell and organelle morphology in silico. Principle of analyzing organelle position using intensity, i.e., perinuclear index; *I*_p_…intensity within a perimeter equidistant to the nucleus, *I*_t_…intensity outside of the perimeter (**A**) or via measuring the distance of individually detected lysosomes from the nucleus (**B**). **C** Simulated cell for testing the robustness of organelle positioning measurements (blue: nucleus, magenta: organelles, white: perimeter for intensity ratio, green: cell perimeter). **D**–**F** Measuring the distance of individual organelles is robust to changes in cell size when normalized by Feret’s diameter and intensity-based measurements (**E**, **F**, green line) are more error prone than distance measurements (**E**, **F**, red line). **G**–**I** Distance measurements of individual organelles are robust to fluctuations in background fluorescence. **J**–**L** Measuring the distance of individual organelles is robust to changes in organelle size

In order to untangle the complex molecular machinery that governs the positioning of organelles, microscopy readouts of pharmacological, genetic, or physiological treatments are essential. When using light microscopy images there are two major means of assessing the relative position of organelles in the cytoplasm: first, by plotting the intensity of a fluorophore which decorates the organelle in relation to a point of reference (Fig. 1A) [38–45](Additional file 1). Second, by detecting or segmenting the organelles and measuring the distance of individual objects to a point of reference (Fig. 1B) [35, 46, 47](Additional file 1). In order to test advantages and disadvantages of these two approaches, we used images of idealized simulated cells consisting of a nucleus, whole cell, and vesicles (Fig. 1C). We measured the integrated density of vesicles in the total cell (*I*_*t*_) and within a defined perimeter equidistant to the nucleus (*I*_*p*_) and calculated the ratio (*I*_*p*_/*I*_*t*_), also known as the perinuclear index (Fig. 1A) [39, 43, 45] as well as the actual distance of the simulated vesicles from the nuclear boundary (Fig. 1B). We then computed the measurement error that the variation of defined morphological parameters would introduce for both methods and dubbed it the *error factor* (see methods section for detailed description). The larger the *error factor* the more sensitive the method is to the morphological parameter.

The positioning of late endosomes and lysosomes (hereafter referred to as lysosomes for simplicity) directly affects cell growth and therefore cell size [48]. We reasoned that altered cell size would skew the results of organelle positioning measurements. Indeed, using our simulated cells we found that altered cell size could introduce a substantial error when performing distance measurements and especially when performing intensity-based quantification (Fig. 1C–E). Normalizing intensity with measurement area decreased the error (Fig. 1F), suggesting that factoring in cell size could at least partially prevent systematic bias when organelle positioning is quantified by intensity-based methods. Normalizing distance by the cell’s diameter (Feret’s diameter) almost completely prevented the error introduced by the altered cell size, regardless of whether cells were oval shaped (Fig. 1F), elongated (Additional file 3: Fig. S1A–D), or cubical (Additional file 3: Fig. S1E–H). Further, basing positioning on intensity measurements is only feasible under the assumption that there is a linear relationship between the intensity of the fluorophore and the occurrence of the organelle. However, uneven distribution of background signal due to inherently low *z*-resolution in light microscopy as well as staining artifacts could alter the result erroneously (Fig. 1G–I). In contrast, when using distance measurements of individual organelles, the influence of the background signal and possible staining artifacts may be eliminated (Fig. 1I). Additionally, organelle size (Fig. 1J–L) or organelle intensity changes due to an experimental treatment cannot be accounted for in intensity-based analysis (e.g., interference with endo-lysosome homeostasis frequently results in vesicle swelling or altered acidity, which would affect the intensity of frequently used endo-lysosomal markers such as lysotracker dyes). Finally, only by detecting individual organelles potentially different subpopulations of organelles can be identified and analyzed further. However, there are limitations to spot detection algorithms used for detecting individual organelles: Detecting sheet-like or crowded organelles may not be faithful (Additional file 3: Fig. S1I–K). In such cases, intensity-based organelle positioning measurements should be the preferred method.

Together, our modeling approach suggests that segmenting individual organelles combined with cell size normalization is the most robust and faithful quantification method for organelle positioning if spot detection can be applied. Based on these results we developed a versatile ImageJ plugin with adjustable parameters for detection and distance measurements of individual organelles as well as intensity distribution measurements in single cells.

### Interactive segmentation and detection using OrgaMapper

**Fig. 2:**
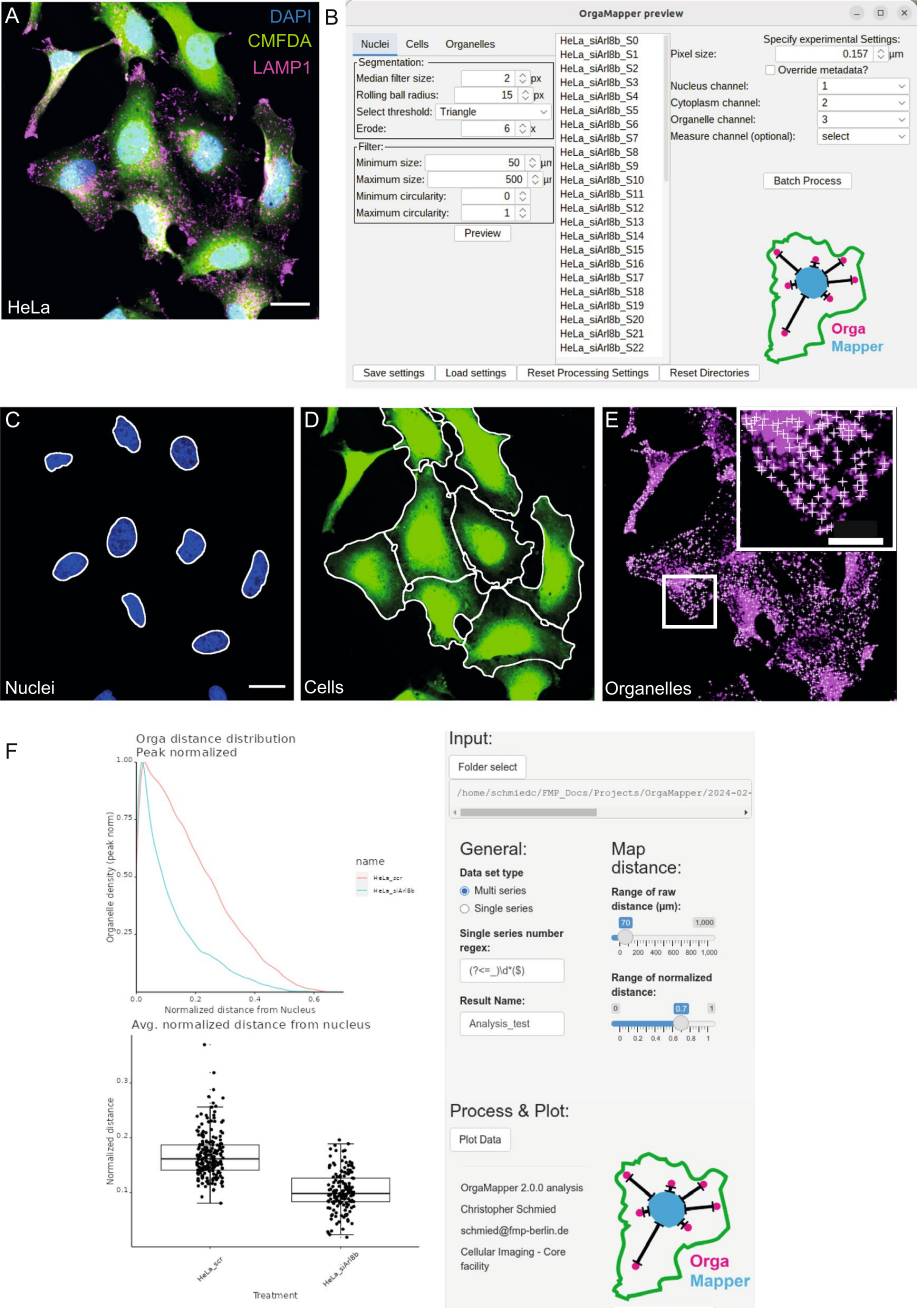
OrgaMapper enables interactive adjustment of segmentation and detection. **A** Typical input data with labels for nuclei (DAPI), cytoplasm (CMFDA), and organelle channel (LAMP1, i.e., lysosomal marker); scale bar 20 µm. **B** The user can adjust and test the segmentation and detection parameters in the GUI of OrgaMapper on all the available files and execute the image analysis in batch. **C** Segmentation of the nucleus; scale bar 20 µm. **D** Cell segmentation filtered for segmented nuclei. **E** Organelle detection excluding organelles overlapping with the nucleus; scale bar in inset 10 µm. **F** OrgaMapper R Shiny graphical user interface

In typical cellular imaging approaches, different subcellular components such as the nucleus, cytoplasm, and the compartment of interest are labeled with appropriate stains or fluorescent markers that report cellular and intracellular architecture (Fig. 2A). To assess the resulting phenotype of the treatment, the labeled structures need to be segmented and measured automatically in a robust manner.

OrgaMapper implements the necessary image processing and analysis tasks for 2D images in one convenient ImageJ plugin. The segmentation can be adjusted via a GUI that allows users to easily and interactively adjust and review the segmentation and detection over the entire dataset and execute the image analysis in batch (Fig. 2B). For reviewing the performance of the image analysis the segmentation and detection region of interests (ROIs) are provided as overlays over the different image channels as preview (Fig. 2C–E). All the necessary analysis parameters are documented and saved in a settings file that can be loaded by the plugin for reproduction. The nuclei are segmented using an automatic intensity-based threshold (Fig. 2C). Nuclei at the edge of the field of view, as well as nuclei that are too small, too large, or irregularly shaped are rejected. For the separation of single cells and the segmentation of the cell area, a marker-controlled watershed is employed. Cells with inappropriate size or shape, without a valid nucleus segmentation, or with multiple nuclei are rejected from further analysis (Fig. 2D). Images in which a plasma membrane stain instead of a cytoplasm stain demarcates single cells, the plasma membrane channel can be used for single-cell segmentation in the same workflow by simply inverting the input channel (Additional file 3: Fig. S2A–C). Single-cell segmentation with OrgaMapper proved faithful for cells of various shapes such as cuboidal epithelial-like cells (Additional file 3: Fig. S2A–C) or elongated fibroblasts (Additional file 3: Fig. S2D–F). In order to robustly detect the peaks of individual organelles within a certain size range an implementation of the Laplacian of Gaussian (LoG) [49] is applied and subsequently, the maxima are detected (Fig. 2E). In addition to the internal segmentation methods, segmentations from other tools and methods such as Labkit [50], ilastik [28], StarDist [51], or cellpose [52] can be loaded if instance segmentation images are provided. External detections can also be loaded if a mask image is provided. The external segmentations and/ or detections can be reviewed over the analyzed dataset in the same manner as the internally generated ones (Additional file 3: Fig. S2G–I).

The segmentation masks of individual nuclei and cells are used to compute for each individual pixel in the cytoplasm the shortest distance to the closest nucleus pixel (i.e., Euclidean distance). This yields a Euclidean distance map (EDM) of the cytoplasm with respect to the edge of the nucleus. The organelle detections are then applied to the EDM within the cytoplasm to extract the location of each organelle in relation to the edge of the nucleus resulting in the generation of a detection map. Additionally, the intensity is measured at the detection localization in the organelle channel. As a further parameter independent from the detection of the organelles, for each pixel in the cytoplasm, the gray value in the organelle channel is extracted together with the distance based on the EDM to generate an intensity map. Distance measurements in OrgaMapper are not limited to the nucleus as a reference point. If desired, distances of detected organelles to the plasma membrane can be obtained. Technically, also other intracellular reference points than the nucleus can be used for distance measurements as long as they can be segmented as a single object per cell, e.g. the microtubule organizing center or the Golgi apparatus (Additional file 3: Fig. S2J). Lastly, OrgaMapper allows for the quantification of radial distributions of organelles by using the center of mass of the nucleus mask as the origin and computing the circular variance of the organelles in reference to the origin (Additional file 3: Fig. S2K–N).

The analysis also extracts per-cell key morphological parameters such as diameter and area as well as overall statistics such as the number of detections, the average intensity in the organelle channel within the entire segmented cytoplasm, and the nuclear center of mass. To estimate a background value, the mean intensity per field of view outside of the unfiltered cell segmentation is measured (Additional file 4). Finally, individual nuclei, cell, and detection ROIs are saved and made accessible for further analysis.

After execution of the Fiji plugin, the analysis results can be loaded into the provided R Shiny App, which automatically collects and saves all measurements in a summary table in the specified output directory. A basic analysis with plots based on the measured parameters is provided by default and visualized in the plotting interface of the R Shiny App. Key settings for the data processing (i.e., background subtraction, cell diameter filter) and plotting (e.g., plotting ranges and bin width) can be adjusted via a GUI (Fig. 2F).

### Spatial statistical analysis enables accurate measurement of organelle positioning phenotypes

**Fig. 3:**
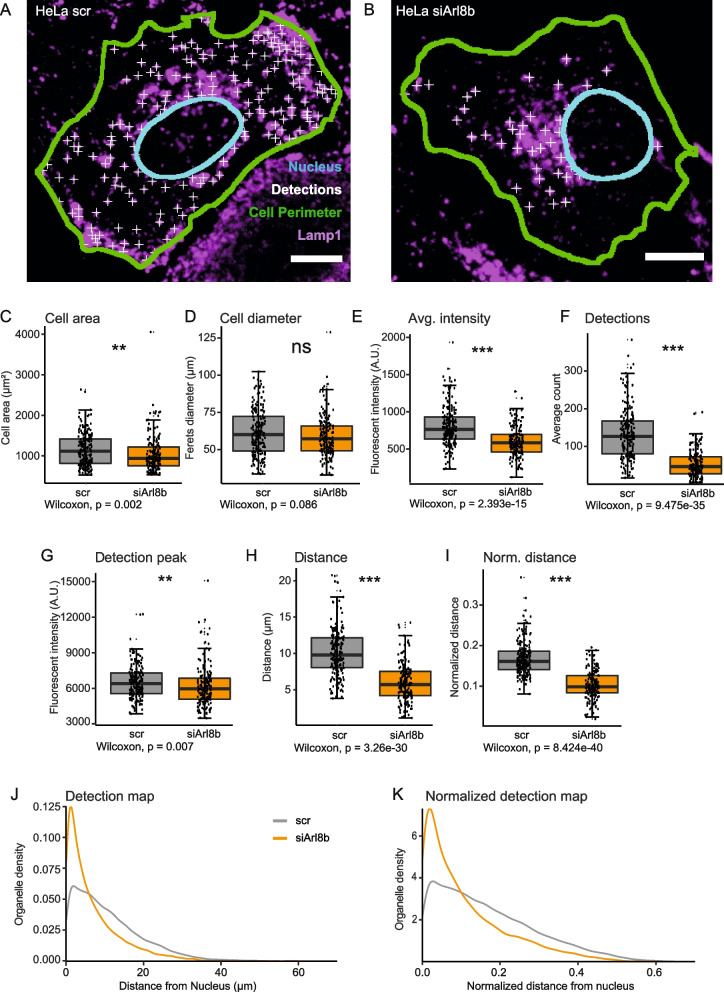
Lysosome positioning analysis with OrgaMapper. **A**, **B **Lysosome staining overlaid with the outline of segmented nuclei and segmented cells (green line) in HeLa cells transfected with scrambled control siRNA (scr) (**A**) or siRNA targeted against Arl8b (siArl8b) (**B**); scale bar 10 µm. Lysosome detections are indicated by white crosses. OrgaMapper analysis extracts parameters such as **C** cell area, **D** cell diameter, **E** average intensity of the organelle channel, **F** number of detections, and **G** organelle intensity at the detection peak. **H**, **I** The analysis shows a significant reduction in the raw distance as well as normalized distance from the nucleus in Arl8b-knockdown cells. **J**, **K** Organelle density plots to visualize an increase in perinuclear lysosomes in knockdown cells in detail (**A**, **B**)

We validated the image and data analysis workflow using a well-known lysosome positioning phenotype that is elicited by depletion of the small GTPase ADP-ribosylation factor-like 8b (Arl8b). It was previously shown that Arl8b loss of function leads to clustering of lysosomes close to the nucleus [48, 53]. Consistently, we observed perinuclear clustering of lysosomes in HeLa cells depleted of endogenous Arl8b by specific small interfering RNA (siRNA) (Fig. 3A, B). Using the OrgaMapper workflow, we could faithfully segment individual HeLa cells and their nuclei and detect individual lysosomes within their cytoplasm (Fig. 3A, B). The image and data analysis also enabled the assessment of general cellular parameters such as cell area, diameter, number of detected lysosomes, the average intensity of the organelle channel within the cytoplasm, and the average gray value at the detection peak (Fig. 3C–G). The measurements yielded a highly significant reduction in the average lysosomal distance with respect to the nucleus in Arl8b-depleted cells, as expected (Fig. 3H). Distance measurements can be normalized to the measured Feret’s diameter of each cell, thereby reducing any measurement error due to variability in cell size (Fig. 3I). Furthermore, the provided analysis enables a closer analysis of organelle localization by plotting the distribution of detected organelles with respect to the nucleus as a kernel density plot. The distribution analysis showed in more detail that the reduction in the average distance of lysosomes is due to an accumulation of lysosomes close to the nucleus (Fig. 3J, K). Together, these results validate the OrgaMapper workflow as a platform capable of extracting comprehensive parameters from microscopy images and faithfully detecting alterations in lysosome positioning. Importantly, accurate detection of single organelles is critical to omit false negatives. Major constraints for detection are the resolution of the microscopy setup (Additional file 3: Fig. S3A–C), the chosen parameters for the LoG filter and the detection noise (Additional file 3: Fig. S3D–F), and finally the nature of the organelle with sheet-shaped organelles proving impervious for spot detection (Additional file 3: Fig. S1I–J). In such cases, the use of the implemented intensity profile-based analysis of OrgaMapper is the recommended approach (Additional file 3: Fig. S4 A–E). If employed on suitable data, the internal OrgaMapper-based detection methods still yield good performance for simple detection problems (Additional file 3: Fig. S4 F–J) as well as adequate detection performance for harder detection problems (Additional file 3: Fig. S4 K–O). More difficult scenarios can be addressed using more advanced methods and loading them as an external detection in OrgaMapper. We have further provided a small macro that enables the user to validate detection methods quantitatively against manual labels to allow users to select suitable detection methods (see Supplemental Material and user documentation).

### OrgaMapper controls for altered cell size and organelle morphology

**Fig. 4:**
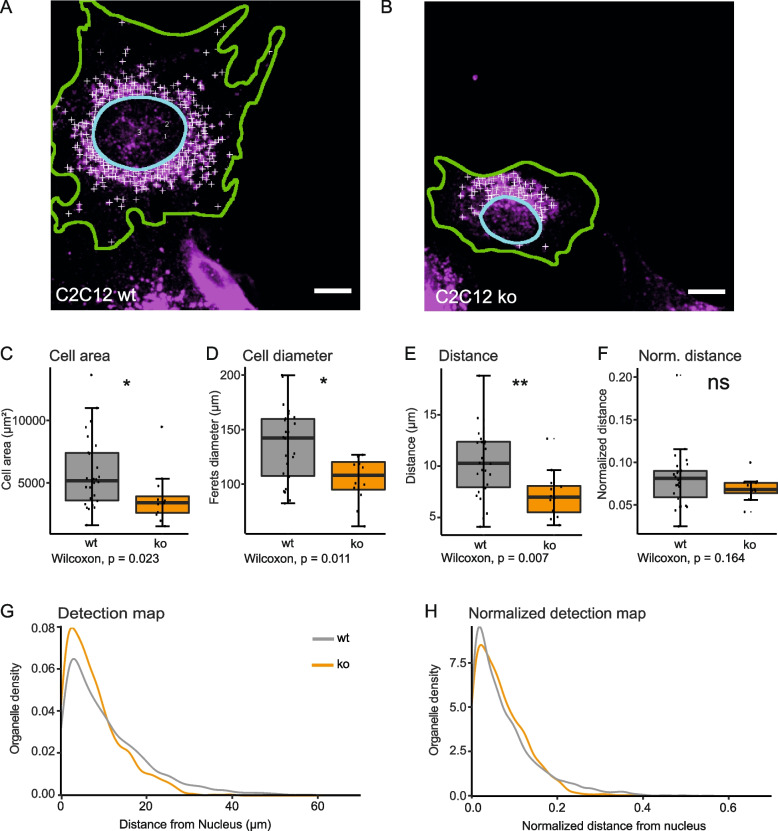
OrgaMapper analysis is robust to changes in cell size. **A** Lysosome staining overlaid with an outline of nucleus (cyan line) and cell segmentation (green line) in C2C12 wild type (wt) and **B** C2C12 MTM1 knockout (ko) cells. Lysosome detections are indicated by white crosses; scale bars 20 µm. **C**,** D** MTM1 knockout significantly reduces cell area and cell diameter. **E** Raw distance measurements indicate a significant reduction of lysosome distance to the nucleus in MTM1 knockout cells. **F** The apparent lysosome positioning change is abolished upon normalization of the data to cell diameter. **G**, **H** Distance maps reveal a lack of difference in lysosome localization when distance measurements are normalized to cell size

OrgaMapper could faithfully detect changes in lysosome positioning, a cellular process that is intimately linked to cell growth [48]. We therefore wanted to validate whether the distance-based OrgaMapper analysis is indeed robust against changes in cell size. To this end, we utilized C2C12 wild-type and MTM1 knockout cells [54]. MTM1 knockout causes a drastic reduction in cell size but no apparent phenotype in lysosome positioning (Fig. 4A, B). As expected, OrgaMapper analysis yielded a significant decrease in cell area (Fig. 4C) and cell diameter (Fig. 4D) in MTM1 knockout cells. Analysis of the raw lysosome distance without cell size normalization suggested a significant reduction in the lysosome distance upon MTM1 knockout (Fig. 4E), contrary to the visual impression. Consistent with the summary statistics, plotting the detailed lysosomal distribution indicated a slight redistribution of lysosomes closer to the nucleus (Fig. 4G). However, normalizing lysosome distance to cell diameter abrogated the difference between wild type and MTM1 knockout cells in lysosome distance measurement (Fig. 4F), and in the normalized distance map (Fig. 4H). These results indicate that disregarding cell morphology can give rise to false positives and demonstrate that the OrgaMapper analysis workflow can correct for such errors.

Another source of error could arise from altered organelle morphology (Fig. 1J–L). In order to test whether OrgaMapper could account for such an error source we used inhibition of the phosphoinositide kinase PIKfyve by the specific inhibitor Apilimod. Apilimod treatment leads to swelling of EEA1-positive endosomes but causes no apparent endosome repositioning (Additional file 3: Fig. S5A, B). OrgaMapper could faithfully detect control and swollen endosomes and found similar distance distributions in both conditions (Additional file 3: Fig. S5C, D).

Together, these results demonstrate that OrgaMapper presents a versatile tool that can compensate for several pitfalls associated with organelle positioning measurements.

### OrgaMapper can map different organelle types and measure associated factors

**Fig. 5:**
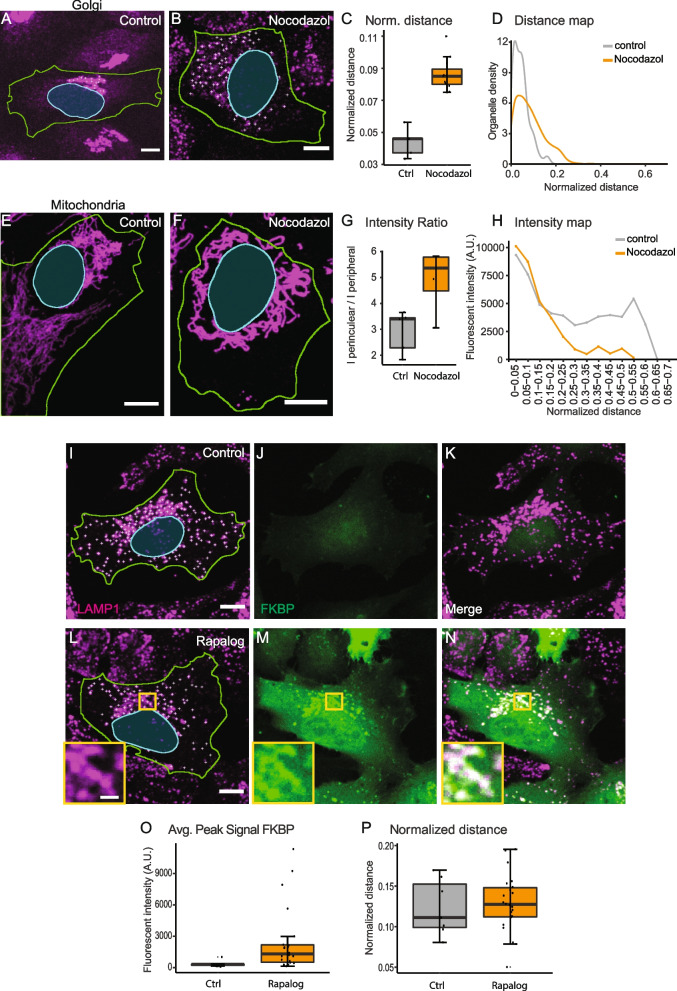
OrgaMapper analysis of different organelle types and intensity measurements of organelle-associated factors. **A**–**D** OrgaMapper analysis performed on control and dispersed Golgi. **A** Scale bar 10 µm. **B** Scale bar 20 µm. **E**–**H** When detections are not possible, such as for Mitochondria, OrgaMapper offers to plot the intensity ratio as well as the intensity distribution from the nucleus; scale bars 10 µm. **I**–**P** OrgaMapper can measure the peak intensity as well as the intensity distribution in an additional channel. **I**–**K** Control cells expressing TMEM192-FRB (dark) and fluorescent protein-tagged FKBP; scale bar 10 µm. **J** do not show enrichment of FKBP on LAMP1-positive structures. **L**–**N** Treatment with Rapalog recruits FKBP to LAMP1-positive structures (**M**) which can be measured with OrgaMapper (**O**). Rapalog treatment does not alter lysosome positioning (**P**). **L** Scale bar in overview 10 µm and 2 µm in inset

Next, we tested whether the OrgaMapper workflow is generalizable and able to assess positioning phenotypes in other organelles than lysosomes. The Golgi apparatus localizes to the perinuclear area via the microtubule organizing center. Microtubule filament disruption by nocodazole disperses Golgi fragments into the cytoplasm (Fig. 5A, B). OrgaMapper could readily detect the difference in Golgi distribution between control and nocodazole-treated conditions (Fig. 5C, D). The dynamics and distribution of mitochondria are dependent on their anchoring to the microtubule cytoskeleton. Consistently, nocodazole treatment causes mitochondria to cluster in the perinuclear area (Fig. 5E, F). In order to analyze mitochondria, OrgaMapper offers plots of intensity ratios or intensity maps that faithfully detected the nocodazole-induced perinuclear clustering of mitochondria (Fig. 5G, H). These collective results demonstrate that the OrgaMapper workflow presents a powerful tool to map the distribution of various organelles.

Organelle function and plasticity are dependent on dynamic re-localization of protein factors between the cytoplasm and the organelle membrane. For instance, dynamic Arl8b localization to lysosomes affects their intracellular distribution (Fig. 3). It is therefore important to examine protein abundance and localization in conjunction with organelle distribution. To enable multiplexed measurements, we extended the analysis modalities of OrgaMapper for intensity measurements in an additional channel. As a proof of principle, we utilized chemically-induced heterodimerization to enrich mRFP-FKBP on lysosomes using an FRB-tagged version of the lysosomal protein TMEM192 and Rapalog, which dimerizes FKBP and FRB (Fig. 5I–N). Recruitment of mRFP-FKBP to lysosomes by Rapalog increased mRFP intensity on lysosomes without affecting lysosome distribution (Fig. 5O, P), exemplifying that OrgaMapper can be used to measure the abundance of organelle-associated factors.

Together, these results demonstrate that the multimodal OrgaMapper architecture enables the assessment of the distribution of multiple organelles together with their associated factors.

## Discussion

In recent years, positioning of cellular organelles in the cytoplasm has garnered substantial research interest from cell biologists and also clinicians. Each individual organelle employs a host of factors to ensure its correct positioning and the adaptation thereof should circumstances demand plasticity. However, the molecular mechanisms controlling organelle positioning dynamics remain incompletely understood. To facilitate the discovery of such pathways and their molecular components, robust, faithful, and scalable analysis pipelines are key. Moreover, for researchers and clinicians who choose organelle positioning as an experimental or diagnostic readout easy-to-use and reliable tools are much needed. Here, we introduce OrgaMapper, an organelle positioning image and data analysis tool that is freely available, easy to use without in-depth image analysis knowledge, and which requires freeware only, that is, Fiji and R. Moreover, OrgaMapper generates informative graphs for all measured parameters and the various means of mapping organelle and intensity distributions.

OrgaMapper is capable of analyzing organelle positioning in 2D microscopy images based on the intensity distribution of an organelle marker or based on the detection of individual organelles. We tested these two approaches for robustness and faithfulness in reporting the distribution of lysosomes. We found that organelle positioning is surprisingly non-trivial to measure and, dependent on the method, can easily give rise to false positives and false negatives.

Using model cells and real-world specimens, we show that detection of individual organelles in combination with cell size normalization is more reliable as compared to widely used intensity-based approaches (Figs. 1 and 4). However, the detection algorithm implemented in OrgaMapper is customized for detecting vesicular structures and may not perform well in detecting organelles with tubular or sheet-like structures such as the mitochondria or the endoplasmic reticulum (Additional file 3: Fig. S4A–E). It is therefore advisable to resort to intensity-based organelle mapping if detection quality is insufficient (Additional file 3: Fig. S4C–E). While the implemented spot detection performance is comparable to similar and state-of-the-art detection methods (Additional file 3: Fig. S4F–O), other limitations of the algorithm are that vesicles with vastly different sizes or staining intensities may not be detected equally well. Likewise, sparse vesicles may be detected more faithfully than heavily crowded ones. To inform the user about the detection quality we implemented the detection preview in the Fiji plugin (Fig. 2E). As further guidance for users we demonstrate the impact of optical resolution (Additional file 3: Fig. S3A–C), explore the parameter space in spot detection (Additional file 3: Fig. S3D–F), and show detection of real world and simulated organelles of various shapes (Figs. 3, 4, and 5, Additional file 3: Fig. S1I–K). An additional macro provided to the user further allows to quantitatively assess the performance of available detection methods. Finally, choosing positive and negative controls and reviewing the detection quality should help the user to decide on whether a detection approach is feasible or whether resorting to intensity-based quantification of organelle positioning is required. To further enhance the versatility of OrgaMapper, segmentation and/or detection results from other external methods can be loaded into the workflow. To enable the detection of non-vesicular organelles future versions of OrgaMapper may include particle detection algorithms based on sophisticated automated intensity thresholds or pre-trained AI models.

We found that organelle positioning measurements are particularly sensitive to cell size. It is well known that mechanisms controlling the positioning of lysosomes are entangled with cell growth signaling pathways [48, 55]. It is therefore advisable to normalize lysosome positioning data by cell diameter, which is, however, not straightforwardly possible with intensity-based quantification strategies such as the widely used perinuclear index. Faithful measurement of the cell diameter relies on faithful detection of the cell edges. A prerequisite for the implementation of OrgaMapper is therefore good quality staining and image quality that is consistent among all compared samples. Future versions of OrgaMapper may implement deep learning models for single-cell segmentation [51, 52]. Applying such a tool may abrogate the need for whole cell stains or nuclei and could free up acquisition channels for other uses such as multiple organelle stains.

## Conclusions

We introduce a novel, easy-to-use, versatile, and robust organelle positioning image and data analysis workflow dubbed OrgaMapper. We validated the workflow using different cellular and organelle models and tested different quantification strategies. In particular, we demonstrated that measuring the distance of individually detected organelles from the nucleus edge is robust to changes in cell size, a common side effect when altering organelle positioning. OrgaMapper can be easily applied as a diagnostic tool or in low to high-throughput microscopy-based screens to identify regulators of organelle positioning and is well-suited as a validation approach for high-content screening assays such as cell painting [56].

## Methods

### Organelle positioning simulation

The robustness of different organelle positioning quantification methods was tested in Fiji using in silico generated 3-channel images containing a single cell comprised of nucleus, cytoplasm, and organelles with defined morphology and organelle positioning. Specifically, 8-bit 500 × 300 pixel 3 channel images were generated in Fiji, region of interests of defined shape and size and flood fill functions were used to generate emulations of nuclei, cytoplasm, and organelles. All images, parameters, and the Macro used to generate the images are available for download (https://doi.org/10.5281/zenodo.10932803). We then made incremental changes to only a single parameter (e.g. cell size) and tested how the parameter affects the result of the organelle positioning quantification using OrgaMapper. Organelle positioning was measured in the simulated cells by two methods: 1) The ratio of integrated density outside of a perimeter with a defined distance to the nucleus over whole cell organelle intensity (i.e., intensity-based) and 2) the average distance of maxima detections to the nucleus (i.e., distance-based). An error factor was computed that relates a given cell parameter (i.e., cell size) to the sensitivity of the measurement results on the parameter. The error factor is computed as fold change of a given result relative to the result obtained in the cell generated with the starting parameter. For starting parameters the error factor was set to 0, i.e., the larger the error factor the more sensitive the measurement is to a given parameter.

### Cell culture

HeLa and U2OS cells were purchased from ATCC. All cell lines were maintained in DMEM containing 4.5 g/ml glucose and L-glutamine (Gibco) supplemented with 10% heat-inactivated fetal calve serum (Gibco) and 100 U/ml penicillin and 100 μg/ml streptomycin (Gibco). Cells were routinely tested for Mycoplasma contamination and all tests were negative. MTM1 knockout C2C12 cell generation was described recently [54]. C2C12 cells were incubated in Ringer’s solution pH 7.4 for 2 h before fixation. MDCKII quintuple claudin knock-out (qKO) cells [57] were provided by the lab of Prof. Mikio Furuse.

### Molecular cloning and expression constructs

For TMEM192-FRB, human TMEM192 and FKBP-rapamycin-binding (FRB) domain of human mechanistic target of rapamycin (mTOR) were amplified by PCR and subcloned into pEGFP/N3 vector from Clonetech by replacing EGFP.

For mRFP-FKBP, mRFP and human FK506-binding-protein 12 (FKBP) were subcloned into pcDNA3.1( +). A pLIB-CMV-FLAG-Cldn16-Puro construct was generated by amplification Cldn16 from phuCldn16-C1 via PCR and replacement of GFP in pLIB-CMV-GFP-N1-Puro [58] via restriction with AgeI and NotI.

### Transfection and gene knockdown

Expression plasmids were transfected into cells using Lipofectamine2000 according to the manufacturer’s instructions. For siRNA transfection, 2.5 × 10^5–3 × 10^5 cells were seeded into 6-well dishes, reverse transfected with 50 nM Arl8b-directed siRNA (GAUAGAAGCUUCCCGAAAU) or scrambled control and 6 µl INTERFERin prediluted in serum-free medium, again transfected forward on day two and analyzed on day four.

Stable FLAG-Cldn16 expressing MDCKII qKO cells were created as described previously [58].

### Immunofluorescence

Cells were seeded on 24-mm coverslips, cultured overnight, treated with indicated reagents, incubated with 5 µM cell tracker green CMFDA for 1 h, fixed 15 min at room temperature with 4% PFA/4% sucrose in PBS, washed 3 × 5 min with PBS, incubated with blocking buffer 1 h room temperature, incubated with overnight at 4 °C with primary antibodies diluted in blocking buffer (see below), washed 3 × 5 min with PBS, incubated 1 h room temperature with fluorophore-conjugated secondary antibodies in 10% goat serum/0.05% Saponin in PBS plus DAPI, washed 3 × 5 min with PBS, mounted on ImmuMount (Shandon), and cured at room temperature.

Blocking buffer for LAMP1 (Lysosomes), EEA1 (Early Endosomes), and GM130 (Golgi) staining was 0.05% Saponin and 10% goat serum in PBS; for TOM20 (Mitochondria) blocking buffer was 0.1% TritonX-100 and 10% goat serum in PBS.

Confluent monolayers of MDCKII qKO cells were fixed with 100% ice-cold ethanol, for 15 min at -20 °C.

### Microscopy

All images were acquired on NikonCSU spinning disc with 40 × air objective (NA = 0.95), or where indicated with 20 × air (NA = 0.75) or 60 × oil (NA = 1.4) objectives, using hardware autofocus and multi-position image acquisition except MDCKII images, which were acquired on a LSM780 (Carl Zeiss Microscopy) with a PL APO DIC M27 40 × /1.3 NA oil objective. Stacks of 7 images with a 1 μm spacing were obtained and maximum intensity projections were created in the Zeiss software.

### Image analysis

Implemented as an ImageJ2 plugin in Java [59] and provided via a Fiji update site. The image analysis is separated into three core tasks: segmentation of nuclei, segmentation of cell area, and detection of organelles. The settings of the image analysis need to be fine-tuned for each individual imaging experiment, thus different settings for each individual image analysis component may apply. To ease the usage of OrgaMapper and to promote reproducibility the settings are saved by OrgaMapper into a XML file for each analysis run. This settings file can easily be opened again in OrgaMapper to apply the same settings with minimal effort. For the datasets in this publication the precise settings are provided for download (https://doi.org/10.5281/zenodo.10932803).

#### Nuclei segmentation

The nuclei segmentation is performed on the DAPI channel. First, a median filter was applied to level out in-homogeneities in the nuclei signal without smoothing of the nuclei edge. For background subtraction, a rolling ball background subtraction was applied. To segment the nuclei, an automatic global intensity threshold was applied. Optionally, the segmentation mask can be adjusted using binary erosions. The particle analyzer is used to reject nuclei at the edge of the field of view as well as apply an optional size and circularity filter (Additional file 3: Fig. S6A).

#### Cell segmentation

For cell segmentation, the CMFDA channel was filtered using a median filter. A rolling ball background subtraction was applied to the filtered image. A fixed global intensity threshold was applied to generate binary masks of the cell area. To separate touching cells a marker-controlled watershed was used. First, the signal of the nuclei channel and the CMFDA channel was added together. The composite image was then filtered using a large Gaussian blur. To determine the separation of the touching cells the find maxima algorithm was applied using the segmented particles option. This applies a watershed algorithm based on the intensity values of the combined and smoothed Nuclei and CMFDA channel and results in a binary mask containing the boundaries of touching cells. The cell area mask and the cell boundary mask were multiplied to generate a binary mask with individual cells separated. The cells were further filtered for size and circularity using the particle analyzer option in ImageJ. Further, the cells were filtered if they did not contain a nuclei segmentation or more than one nuclei segmentation (Additional file 3: Fig. S6B).

#### Organelle detection

To detect individual blob-shaped organelles the organelle channel was filtered using an ImageJ implementation of the Laplacian-of-Gaussian filter [49]. Individual organelles were then detected using a maxima detection. The detections were filtered for excluding detections in the nuclei mask (Additional file 3: Fig. S6C).

#### Measurements

We extracted key measurements per well such as total cell count and mean intensity of the background based on the area outside of the cell segmentation. For each cell, we further extracted parameters such as cell area, ferret diameter, and mean intensity of the organelle channel as well as an optional measurement channel within the cytoplasmic area (cytoplasmic area: cell mask minus nuclei mask). To determine the distance from the nucleus of each organelle detection, an EDM was computed per cell, which is a very fast computation as compared to the algorithm used by [35]. For each individual detection within each cell, the distance based on the EDM was extracted (Additional file 3: Fig. S6D). Further, the signal intensity at that location of the detection was measured in the organelle channel as well as an optional measurement channel. As an alternative detection-independent measurement, the distance of each individual pixel within the cytoplasmic mask of each cell was extracted as well as the corresponding intensity value in the organelle channel and measurement channel.

### Statistical analysis and image and data visualization

The results of the image analysis with the Fiji plugin were collected using the OrgaMapper Shiny app. Further, the Shiny app allows basic and advanced descriptive data analysis. Intensity measurements were background subtracted using the background intensity measured outside of the segmented cell area in the organelle and additional measurement channel. For the analysis, a cell diameter can be applied (set to 600 µm). Distance measurements were normalized per cell using the extracted Feret’s diameter of the cell. For visualizing the organelle distribution based on the organelle detection a kernel density plot was computed. The intensity distribution was based on binning the intensity values and visualizing as a line plot. The intensity ratio was computed using a separate R script by dividing the cell into two areas using a fixed perimeter of 10 µm. The mean intensity from the area closer to the nucleus was divided by the mean intensity away from the nucleus. All measured and evaluated cellular and organelle parameters were visualized as box plots overlaid with a dot plot. The statistical significance between control and treatment was evaluated using an unpaired two-sample Wilcoxon rank sum test. Images for visualization were processed using established methods and standards in bioimage analysis [32, 60].

#### Analysis of organelle radial distribution

The center of mass of the nucleus mask is used as origin in the cell. The *x*, *y* location of the organelle detections are computed in reference to this origin by subtracting the *x*, *y* location of the center of mass of the nucleus in the image from the *x*, *y* location of the detection in the image. The arctangent of the detections around the origin was computed using the atan2 function. The resulting radians were mapped back to 0–360°. Circular variance was computed using the circular R package version 0.5.0.

#### Quantitative validation of detection performance

For the quantitative evaluation of the detection performance, we defined two levels of detection difficulty. The easy level encompasses small objects with little variable image background and comparable signal-to-background ratio over the range of examples as well as within the example. The hard detection level encompasses objects with varying intensity to background across the different selected examples and also within the examples. For each detection difficulty level, we selected three example cells that were identified and cropped. The objects in the cytoplasm within each individual cell were labeled and curated manually as a comparison standard. The manual Fiji regions of interest were turned into a binary mask with a single pixel of value 255 marking the point of manual detection.

Automatic detections with the same settings were then applied over the examples in each difficulty level. The internal OrgaMapper detection for the easy examples was applied with a LoG sigma of 2 and a prominence of 100. For the difficult detection challenge, the LoG sigma was set to 1.5 and the prominence to 120. For comparison, another classic detection algorithm based on Wavelet-based detection [61] implemented in Icy version 2.5.2 [27] was used. The easy example was analyzed with the UDWTWaveletDetector set to detect bright spots over a dark background with scale 2 (object size ~ 3 pixels) and a sensitivity of 35. The hard example was analyzed using the UDWTWaveletDetector set to detect bright spots over a dark background with scale 2 (~ 3 pixels) and sensitivity set to 110. Finally, a pre-trained deep learning-based detection method was employed by applying the general model of Spotiflow [62] using its napari plugin [63] on both the easy and hard detection. For each automatic detection binary detection masks were produced with single pixels of value 255 marking the point of automatic detection.

We used a distance metric to evaluate the performance of the automatic detection methods in comparison to the manually curated labels [61]. First, a distance matrix was computed by computing the pairwise distance of all points between manual and automatic segmentation using Euclidean distance:

$$\mathrm{sqrt}\left(\left({\mathrm x}_1-{\mathrm x}_2\right)^2+\left({\mathrm y}_1-{\mathrm y}_2\right)^2\right)$$

*x*, *y*: coordinates of compared points in pixels

This distance matrix image was then thresholded using a value of 4, corresponding to the smallest distance between individual objects. Detections across the compared manual and automatic detection below this threshold are defined as true positive (TP). False positive (FP) detections are defined as detections found only in the automatic detections and false negative (FN) detections only in the manual label. Ambiguous detections (*N*_ambi_) are defined as multiple automatic detections within the minimum distance threshold indicating an over-detection of the method. Based on this, an F1 score was computed using the following formula:

$$\mathrm F1=\mathrm{TP}/\mathrm{TP}+0.5^\ast\left(\mathrm{FN}\;+\;\mathrm{FP}\right)$$

Over all examples within each detection challenge an average F1 score was computed.

## Materials

### Reagents

 The reagents are shown in the table below.

**Table:** 

Name	Company	Product number	Working concentration
Cell tracker green CMFDA	Invitrogen	C7025	5 µM
Nocodazole	Sigma	M1404	20 µM
Apilimod	Sigma	SML2974	1 µM
A/C Heterodimerizer (Rapalog)	Takara	635,095	500 nM
Arl8b siRNA	Sigma		100 nM
Sigma Mission® siRNA universal negative control #1	Sigma	SIC001	100 nM

### Antibodies

 The antibodies are shown in the table below.

**Table:** 

Name	Company	Product number	Dilution
Anti-LAMP1	BD pharmingen	555,798	1:200
Anti-TOM20	Santa Cruz	sc-17764	1:200
Anti-GM130	Abcam	ab52649	1:200
Anti-EEA1	Cell Signaling	2411	1:100
Anti-Calreticulin	Thermo	PA 3–900	1:200
Anti-FLAG	Sigma-Aldrich	F3165	1:200
Anti-rabbit CF647	Biotium	20,047	1:1000
Anti-mouse CF647	Biotium	20,046	1:1000
anti-mouse AlexaFluor488	Invitrogen	A11029	1:1000

## Supplementary Information

Additional file 1. Alternative analysis. Table of alternative analysis approaches with a classification of the automation (manual, semi-automatic, automatic), algorithm category (count ratio, radial intensity, intensity ratio, distance), algorithm description, software platform, availability, usability score, license and methods description

Additional file 2. Workflow score. Usability score of different workflows assessed according to availability of documentation (Docu), test data (Test), how easy the workflow is to adjust (Adjust), easy of install (Install), availability of analysis (Analysis).

Additional file 3. Figures S1-S6. Fig. S1: Measuring the distance of individual organelles from the nucleus is robust to changes in cell size regardless of cell shape but performs weakly with sheet-like organelles. Fig. S2: Segmentation and measurement versatility of OrgaMapper. Fig. S3: Impact of optical resolution, detection parameters, and organelle shape on spot detection quality. Fig. S4. Analysis modality selection based on organelle morphology and quantitative comparison of detection methods. Fig. S5: OrgaMapper analysis is robust to organelle swelling.Fig. S6: OrgaMapper workflow diagram of main modules.

Additional file 4. OrgaMapper measurements. Table listing measured parameters with information provided on measurement type, if available as standard or optional parameter, exact name of variable and a short description of what is measured.

## Data Availability

The image analysis workflow is available as a Fiji plugin and can be installed via an updated site: https://sites.imagej.net/Cellular-Imaging/. The source code of the R Shiny App can be downloaded from GitHub: https://github.com/schmiedc/OrgaMapper_Rshiny. Documentation for the usage of the Fiji plugin and R Shiny App is available here: https://schmiedc.github.io/OrgaMapper/. The software, test datasets, simulation images, the simulation cell generation macro, and settings files can be found in the following zenodo repository: https://doi.org/10.5281/zenodo.12773379 Contact and support: https://forum.image.sc/u/schmiedc/.

## Abbreviations

GUI: Graphical user interface
ROI: Region of interest
LoG: Laplacian of Gaussian
EDM: Euclidean distance map
Arl8b: ADP-ribosylation factor-like 8b
siRNA: Small interfering RNA
qKO: MDCKII quintuple claudin knock-out
FRB: FKBP-Rapamycin-binding
mTOR: Mechanistic target of rapamycin
FKBP: FK506-binding-protein 12
TP: True positive
FP: False positive
FN: False negative
