# Supplementary material for: OrgaMapper: a robust and easy-to-use workflow for analyzing organelle positioning
Source: BMC Biol. 2024 Sep 30;22:220. doi: 10.1186/s12915-024-02015-8 (PMC11440938; doi:10.1186/s12915-024-02015-8)
Supplement: Supplementary file 3 — Additional file 3. Figures S1-S6. Fig. S1: Measuring the distance of individual organelles from the nucleus is robust to changes in cell size regardless of cell shape but performs weakly with sheet-like organelles. Fig. S2: Segmentation and measurement versatility of OrgaMapper. Fig. S3: Impact of optical resolution, detection parameters, and organelle shape on spot detection quality. Fig. S4. Analysis modality selection based on organelle morphology and quantitative comparison of detection methods. Fig. S5: OrgaMapper analysis is robust to organelle swelling.Fig. S6: OrgaMapper workflow diagram of main modules. [file 12915_2024_2015_MOESM3_ESM.docx]

# Supplemental Data & Figures

## Fig. S1


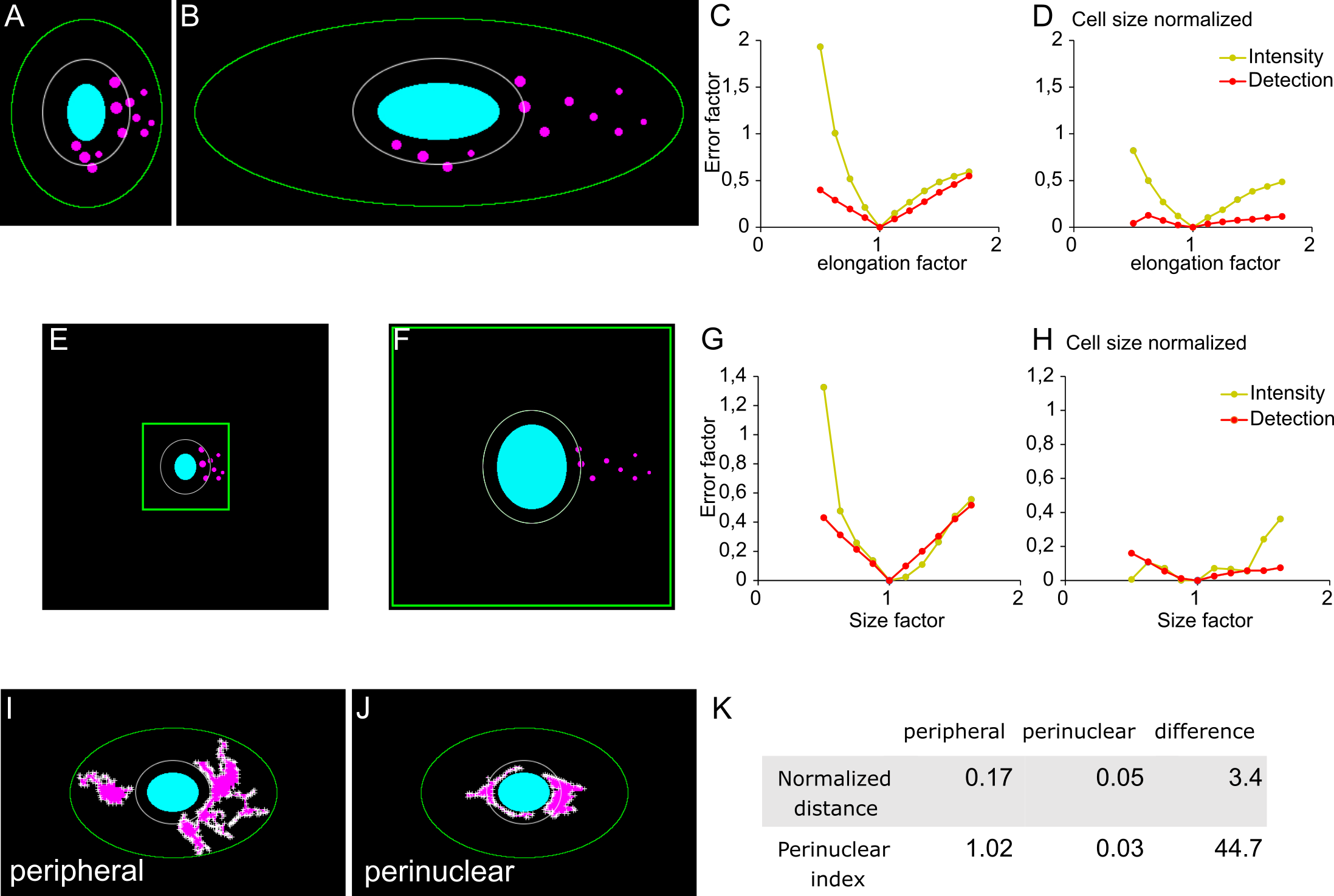


**Fig. S1:** **Measuring the distance of individual organelles from the nucleus is robust to changes in cell size regardless of cell shape but performs weakly with sheet-like organelles.** Simulated cells for testing robustness of organelle positioning measurements (blue: nucleus, magenta: organelles, white: perimeter for intensity ratio, green: cell perimeter). (**A-D**) Measuring the distance of individual organelles is robust to changes in cell elongation when normalized by Feret’s diameter and distance measurements are more robust than intensity distribution measurements. (**E-H**) Measuring the distance of individual organelles is robust to cell size changes in cuboidal cells when normalized by Feret’s diameter. (**I, J**) Simulated sheet-shaped organelles are not readily detectable by spot detection, only organelle edges can be detected (sheet-shaped organelle in magenta, spot detection as white crosses, for spot detection the Laplacian of Gaussian Sigma parameter was set to a minimal value to enable detection of organelle edges). (**K**) Spot detection-based distance measurements of peripheral versus perinuclear sheet-shaped organelles underestimate the positioning difference (3.4-fold) as compared to a perinuclear index (44.7-fold difference).

## Fig. S2.


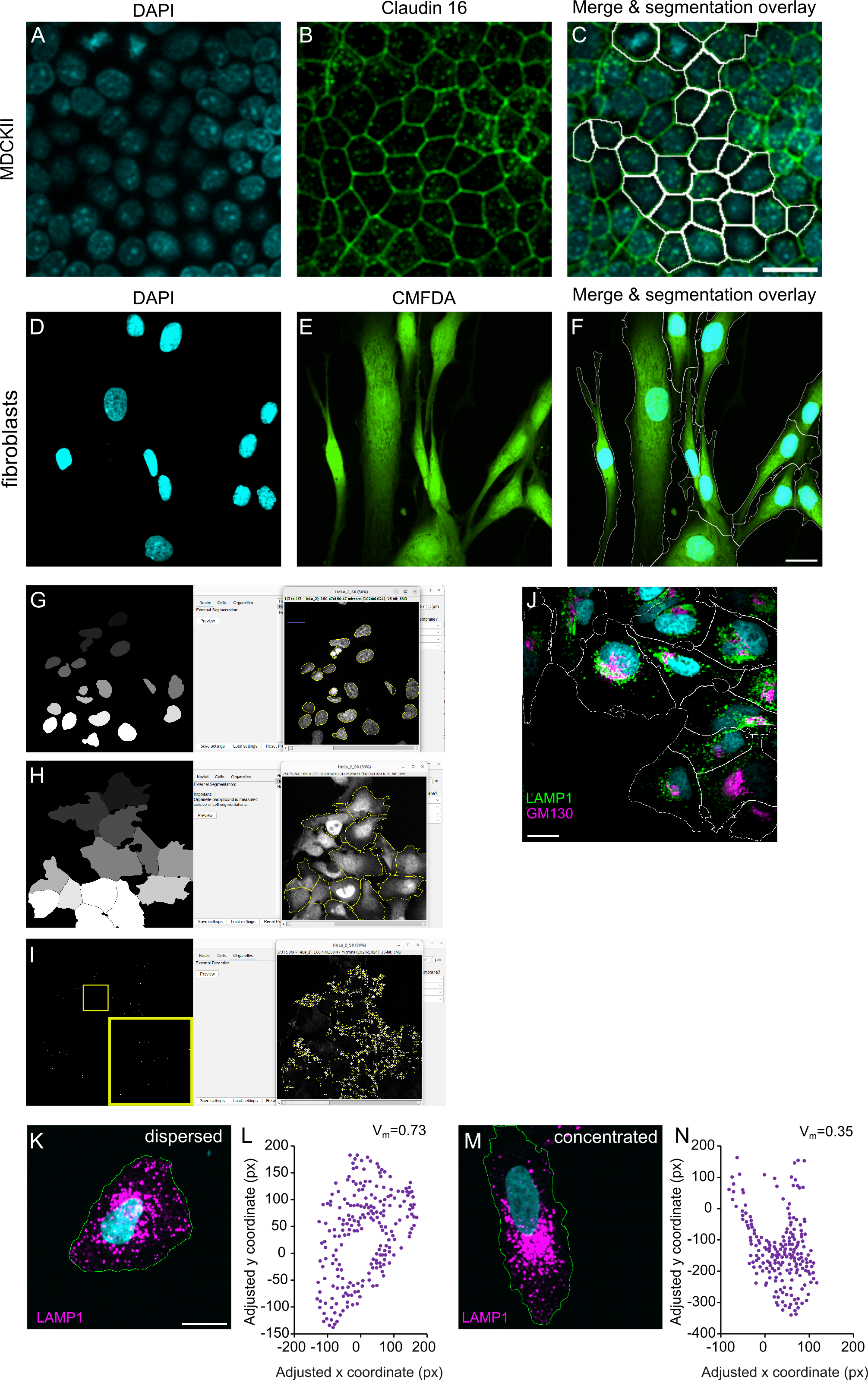


**Fig. S2:** **Segmentation and measurement versatility of OrgaMapper.** (**A-D**) Fixed MDCKII cells expressing FLAG-tagged Claudin 16 stained with DAPI to visualize the nucleus (**A**) and antibodies directed against FLAG to visualize the plasma membrane (**B**) were segmented in OrgaMapper (**C**) using the *Invert Cell Image* option; cell outlines are shown in white, scale bar 20 µm. Fibroblasts were stained with DAPI to highlight the nucleus (**D**), CMFDA to highlight the cytoplasm (**E**), and segmented with OrgaMapper (**F**), cell outlines are shown in white, scale bar 20 µm. (**G-I**) Nucleus segmentation, cytoplasm segmentation, and organelle detection from external modalities can be imported into OrgaMapper. (**J**) OrgaMapper allows for single cell segmentation and distance measurements using the Golgi apparatus as reference point: Hela cells were fixed and stained with DAPI (cyan) and antibodies against the lysosomal protein LAMP1 (green) and the Golgi protein GM130 (magenta); single cells were segmented in OrgaMapper using the GM130 channel as nucleus channel; cell outlines, Golgi outlines and LAMP1 detections are shown in white, scale bar 20 µm. (**K-N**) OrgaMapper enables the analysis of the radial distribution of organelles. U2OS cells were fixed, stained for nuclei with DAPI (cyan), LAMP1 for lysosomes (magenta), and CMFDA for cytoplasm (not shown), and analysed by OrgaMapper; cell outlines are shown in green (**K, M**); individual detections were plotted according to their position with respect to the nuclear center of mass and the circular variance (V_m_) was computed in R (**L, N**).

## Fig. S3

##


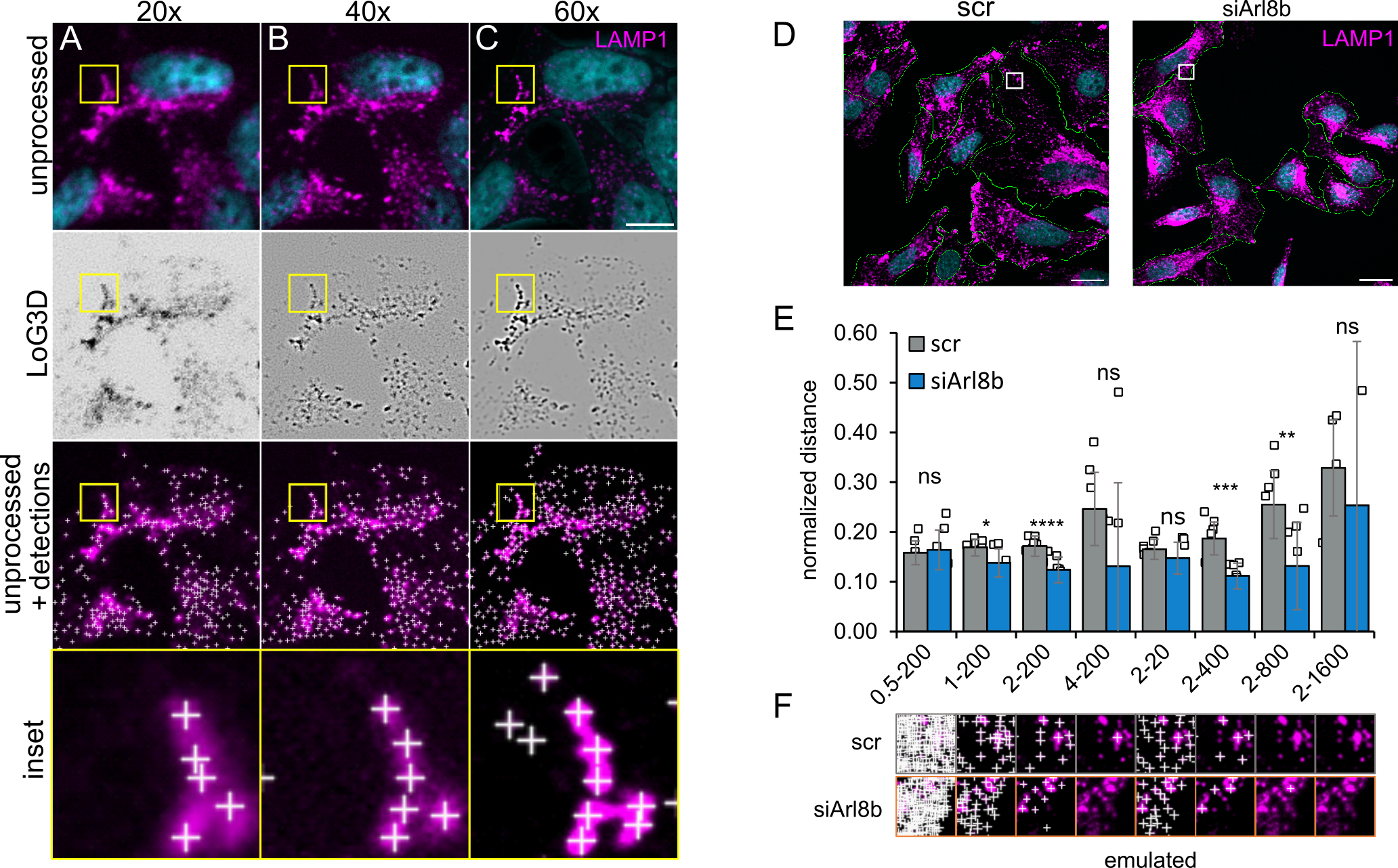


**Fig. S3:** **Impact of optical resolution, detection parameters, and organelle shape on spot detection quality.** (**A-C**) HeLa cells were fixed and stained with DAPI (cyan) and antibodies directed against LAMP1 (magenta) to visualize nuclei and lysosomes, respectively; the same field of view was imaged with 20x (**A**), 40x (**B**), and 60x (**C**) objectives and analyzed by Laplacian of Gaussian (LoG 3D) filtering (second row, grey) and spot detection (third row and fourth row for high magnification insets in yellow, detections in white); scale bar 10 µm. (**D**) HeLa cells were transfected with scrambled control (scr) siRNA (left panel) or siRNA directed against Arl8b to induce perinuclear clustering (right panel), fixed, and stained with DAPI (cyan) and antibodies directed against LAMP1 (magenta) to visualize nuclei and lysosomes, respectively, and segmented in OrgaMapper, cell outlines are shown in green, scale bars 20 µm. (**E**) Choosing inadequate spot detection parameters can give rise to false negatives: Lysosome positioning in cells shown in (**D**) was analyzed by distance measurements in OrgaMapper using a range of filtering and detection parameters (number before hyphen in x-axis labelling refers to *LoG Sigma*, number after hyphen refers to *Prominence*), ns…non significant, * p<0.05, ** p<0.01, *** p<0.001, **** p<0.0001. (**F**) Detections of lysosomes in inset boxes in (**D**) generated with parameter combinations shown in (**E**).

## Fig. S4


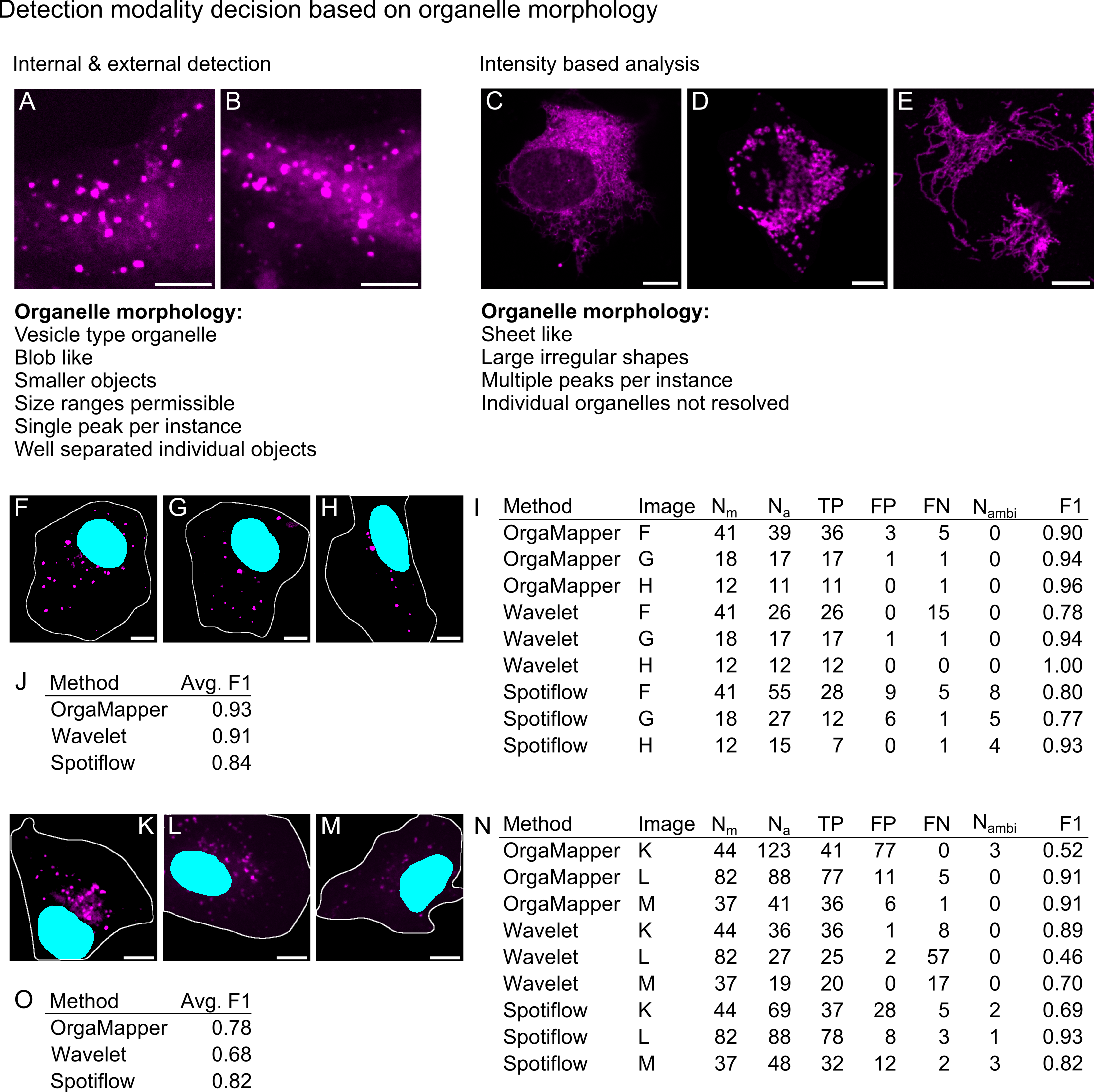


**Fig. S4. Analysis modality selection based on organelle morphology and quantitative comparison of detection methods.** (**A-E**) The analysis strategy of OrgaMapper is flexible in regard to the organelle morphology. For vesicle type organelles with discreet blob-like objects, which can be separated from the background, an internal as well as externally provided detection methods can be selected; scale bar 10 µm, (**A-B**). Note that image (**A**) and (**B**) are crops of (**F**) and (**K**) respectively as they server as illustrations of an easy and difficult detection example. For organelles that do not fit the detection paradigm such as organelles with sheet-like morphology (GFP-Rab18, C), organelles with multiple peaks per instance (LAMP1 vacuoles induced by Apilimod, **D**), or tube-like morphologies (Mitochondria visualized by antibodies against Tom20, **E**) the intensity-based analysis can be employed; scale bar 10 µm (**C-E**). (**F-J**) Quantitative comparison of automatic detection methods with manual detection in images with easily detectable organelles, i.e. images with well separated objects of comparable intensity and little background in the cytoplasm; scale bar 10 µm (**F-H**). In each cell organelles were labeled manually (N_m_) and automatic detections (N_a_) were performed with the same settings on all examples using the internal OrgaMapper detection (OrgaMapper), a wavelet based detection (Wavelet) as well as a pre-trained model of a deep learning based detection method (Spotiflow); each row represents results of one cell. The number of true positive (TP), false positive (FP), false negative (FN) and ambiguous detections (N_amb_) was determined to compute an F1 score (**I**). The average F1 score (Avg. F1) in the easy detection paradigm revealed that detection methods are able to perform with similar adequate performance. The lower performance of the deep learning-based detection method, producing more FP and N_amb_ detections, is likely the result of the use of a pre-trained model that was not optimized towards the specific detection problem. A properly re-trained network would likely have a much better performance (**J**). (**K-O**) Quantitative comparison of the detection performance on a more difficult detection problem, i.e. organelles with varying intensity per example and elevated background; scale bar 10 µm (**K-M**). The performance of the internal detection method as well as the wavelet-based detection method is decreased compared to the easy detection challenge with an increase of FP and FN detections (**N**) and corresponding lower average F1 scores. The performance of the deep learning-based detection method is more robust, likely because its detection performance is not as sensitive towards signal variance across and within the examples (**K**).

## Fig. S5

##


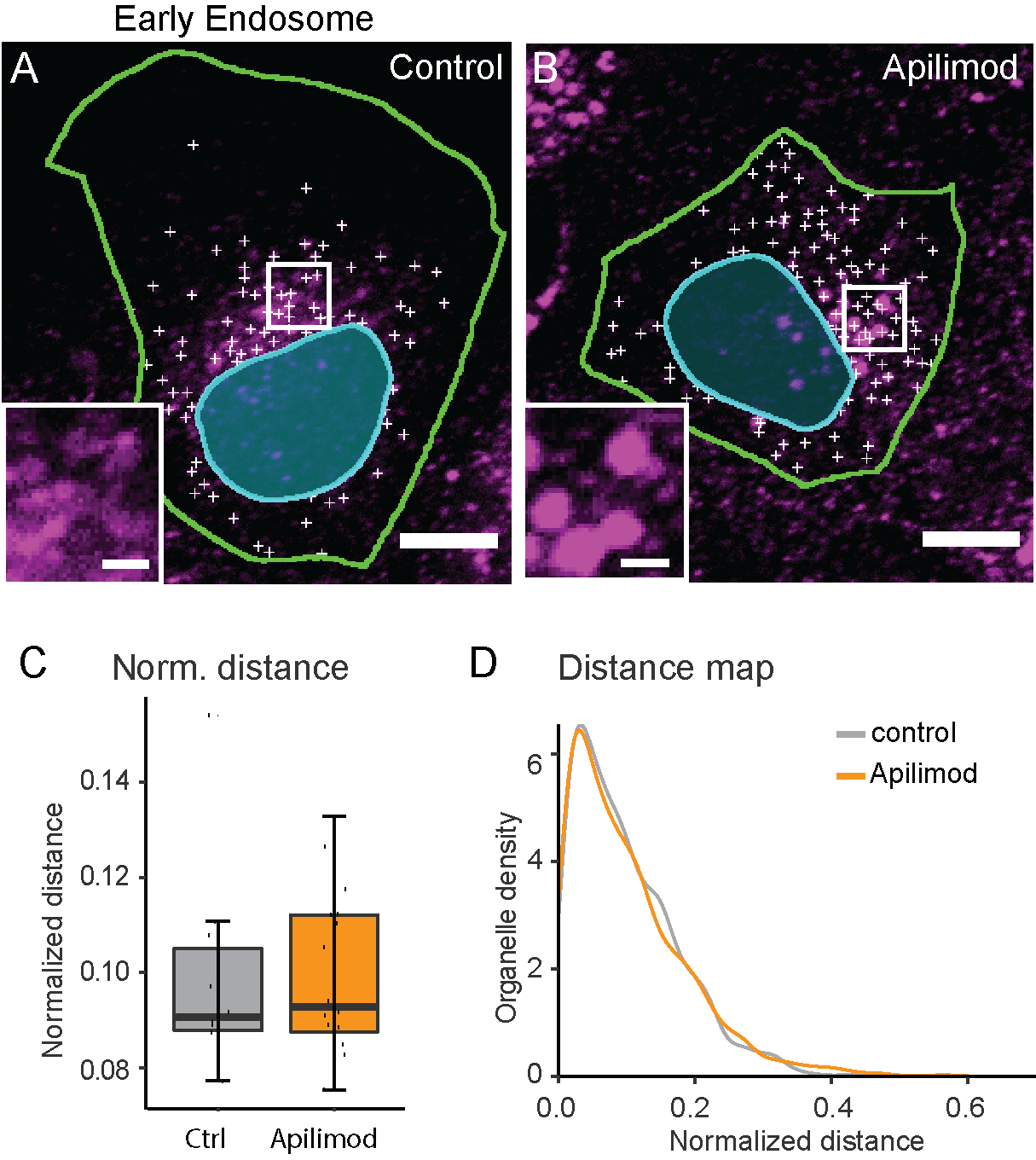


**Fig. S5: OrgaMapper analysis is robust to organelle swelling**. OrgaMapper analysis performed on early endosomes marked by an EEA1-specific antibody in control (**A**) and Apilimod treated conditions in which endosomes undergo swelling; scale bar in overview 10 µm; scale bar in inset 2 µm (**B**). Normalized distance of detected endosomes to the nucleus (**C**) and distance mapping (**D**) do not show a difference in organelle positioning.

##
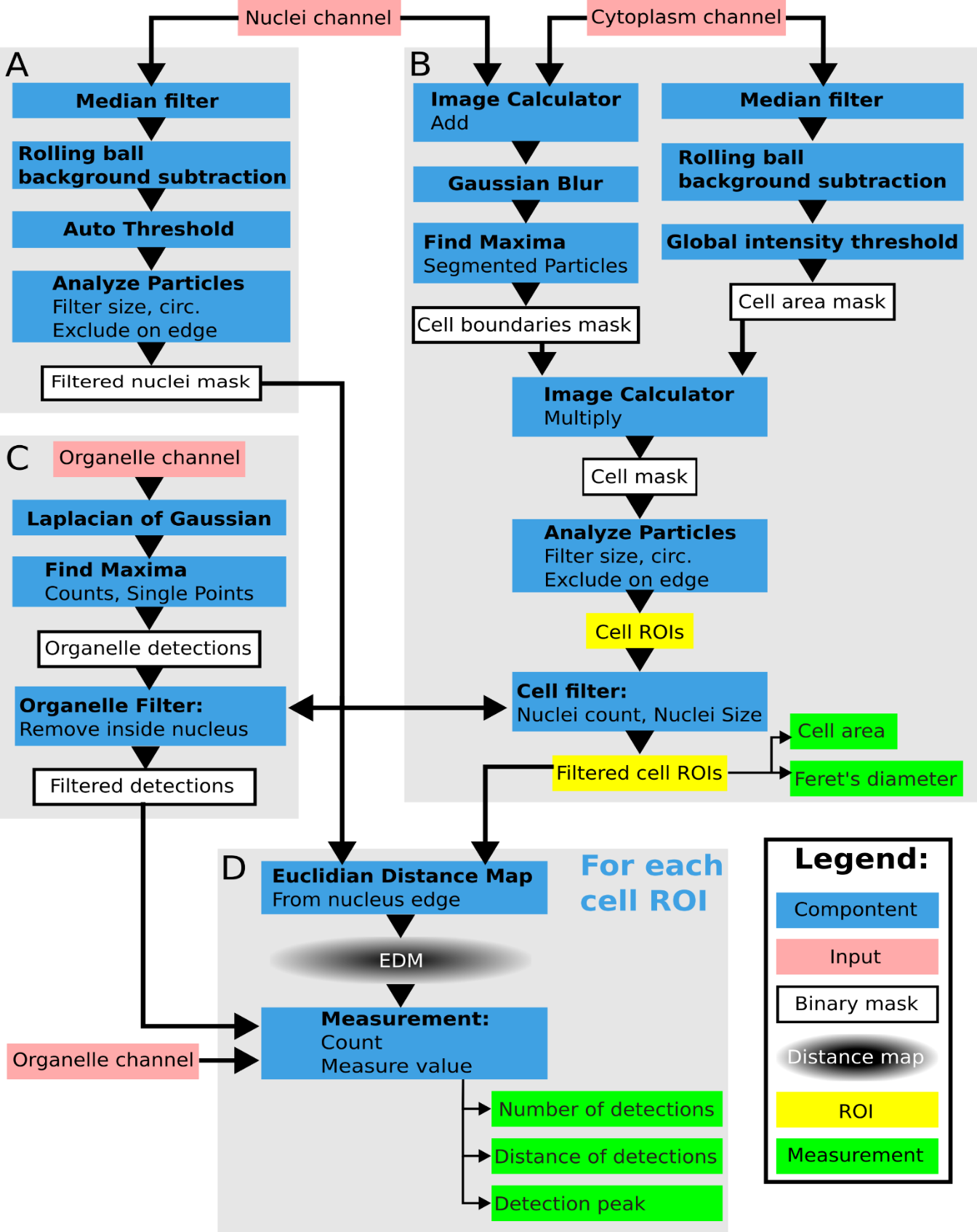
Fig. S6

**Fig. S6: OrgaMapper workflow diagram of main modules.** (**A**) Nuclei segmentation. (**B**) Cell segmentation. (**C**) Organelle detection. (**D**) Organelle measurement.
